# Pseudogenization of a resistance-nodulation-division (RND) efflux pump subunit underlies drug and cell envelope stress sensitivity in Brucella ovis

**DOI:** 10.51894/001c.143477

**Published:** 2025-10-01

**Authors:** Thomas Kim, Bill Hong, Erika Lisabeth, Richard Neubig, Aretha Fiebig, Sean Crosson

**Affiliations:** 1 College of Osteopathic Medicine Michigan State University

## 01

Brucella species are intracellular bacterial pathogens that pose a significant threat to both animal and human health. To identify compounds that reduce Brucella ovis fitness in mammalian phagocytes, we conducted a high-throughput luminescence-based screen, which revealed dihydropyridine-class Ca²⁺ channel blockers nicardipine and cilnidipine as potential host-targeting anti-infectives. However, follow-up dose-response studies in pure culture revealed that these compounds directly inhibit B. ovis growth. To investigate possible dihydropyridine resistance mechanisms, we selected for B. ovis mutants tolerant to these drugs and identified single-base deletions in the bepE pseudogene. These mutations restored a functional open reading frame for BepE, a subunit of a resistance-nodulation-division (RND) efflux pump, increasing B. ovis resistance to dihydropyridine treatment. Given that B. ovis has undergone extensive pseudogenization and exhibits greater chemical susceptibility than other Brucella species, we examined whether bepE influenced cell envelope integrity. B. ovis mutants with an intact bepE gene displayed enhanced resistance to membrane disruptors, including deoxycholate. To extend these findings, we investigated bepE function in Brucella abortus, a closely related zoonotic pathogen that encodes a fully intact BepE protein. Deleting bepE in B. abortus increased its susceptibility to deoxycholate and its sensitivity to cilnidipine during macrophage infection, indicating that bepE not only contributes to drug resistance in the intracellular niche but also supports B. abortus resistance to cell envelope stress. The results define bepE as a determinant of Brucella resistance to antimicrobial compounds, and demonstrate that its pseudogenization contribute to the heightened chemical sensitivity of B. ovis relative to other classical Brucella species.

